# Estimation of Noise-Free Variance to Measure Heterogeneity

**DOI:** 10.1371/journal.pone.0123417

**Published:** 2015-04-23

**Authors:** Tilo Winkler, Marcos F. Vidal Melo, Luiza H. Degani-Costa, R. Scott Harris, John A. Correia, Guido Musch, Jose G. Venegas

**Affiliations:** 1 Department of Anesthesia, Critical Care and Pain Medicine, Massachusetts General Hospital and Harvard Medical School, Boston, Massachusetts, United States of America; 2 Pulmonary and Critical Care Unit, Department of Medicine, Massachusetts General Hospital and Harvard Medical School, Boston, Massachusetts, United States of America; 3 Radiology Cyclotron Laboratory; Department of Radiology, Massachusetts General Hospital and Harvard Medical School, Boston, Massachusetts, United States of America; Glasgow University, UNITED KINGDOM

## Abstract

Variance is a statistical parameter used to characterize heterogeneity or variability in data sets. However, measurements commonly include noise, as random errors superimposed to the actual value, which may substantially increase the variance compared to a noise-free data set. Our aim was to develop and validate a method to estimate noise-free spatial heterogeneity of pulmonary perfusion using dynamic positron emission tomography (PET) scans. On theoretical grounds, we demonstrate a linear relationship between the total variance of a data set derived from averages of *n* multiple measurements, and the reciprocal of *n*. Using multiple measurements with varying *n* yields estimates of the linear relationship including the noise-free variance as the constant parameter. In PET images, *n* is proportional to the number of registered decay events, and the variance of the image is typically normalized by the square of its mean value yielding a coefficient of variation squared (*CV* ^2^). The method was evaluated with a Jaszczak phantom as reference spatial heterogeneity (CV_r_^2^) for comparison with our estimate of noise-free or ‘true’ heterogeneity (*CV*_t_^2^). We found that *CV*_t_^2^ was only 5.4% higher than *CV*_r_^2^. Additional evaluations were conducted on 38 PET scans of pulmonary perfusion using ^13^NN-saline injection. The mean *CV*_t_^2^ was 0.10 (range: 0.03–0.30), while the mean *CV* ^2^ including noise was 0.24 (range: 0.10–0.59). *CV*_t_^2^ was in average 41.5% of the *CV* ^2^ measured including noise (range: 17.8–71.2%). The reproducibility of *CV*_t_^2^ was evaluated using three repeated PET scans from five subjects. Individual *CV*_t_^2^ were within 16% of each subject's mean and paired t-tests revealed no difference among the results from the three consecutive PET scans. In conclusion, our method provides reliable noise-free estimates of *CV*_t_^2^ in PET scans, and may be useful for similar statistical problems in experimental data.

## Introduction

Variance is a valuable statistical parameter for quantifying the heterogeneity or variability of data. However, noise, as random measurement errors superimposed to the signal, contributes to the variance of the measured data. A resulting overestimation in variance can be a substantial problem for accurate assessments of heterogeneity, for fractal analysis, and for comparisons of heterogeneity under different conditions or at different time points.

In biomedical imaging, spatial heterogeneity of an image is typically estimated as variance normalized by the square of the mean, also defined as coefficient of variation squared (CV^2^). Positron emission tomography (PET) imaging as opposed to computer tomography (CT) typically involves higher levels of noise relative to the signal creating a challenge for the assessment of spatial heterogeneity in structure and function that is relevant for our understanding of the normal physiology or of changes caused by diseases. Despite this challenge, the variety of tracers for measuring unique functional and molecular processes in-vivo makes PET a very versatile imaging modality. PET measurements of the functional heterogeneity in organs include for example the heterogeneity of perfusion and ventilation in the lungs [1–6], cerebral perfusion and glucose metabolism in the brain [7], perfusion heterogeneity in human skeletal muscle [8], and glucose metabolism in sarcoma [9]. Additionally, measurements of heterogeneity using PET have been used for fractal analysis of physiological processes [5,10–12] although the estimates of fractal dimension may be biased by the contribution of noise to the variance [13].

The overestimation of spatial heterogeneity in PET scans due to noise can be reduced by filtering of reconstructed images or with filtered backprojection methods, preferred for quantitative image analysis, that were developed to reduce noise [14,15]. But such filter-based approaches are limited by the trade-off between reducing noise and degrading the resolution [15–17]. Some other approaches avoiding this limitation rely on an approximation for the noise [18], or require access to raw image data [14,19–24], which is often limited by proprietary raw data formats. One method suitable for reconstructed PET scans showed a correlation between the coefficient of variation squared and the reciprocal of the average counts per voxel that characterized the noise in PET images of a phantom [25].

Our aim in this study was to identify the theoretical basis of the relationship between variance and reciprocal of counts, and to use the relationship in dynamic PET scans for estimation of noise-free, or ‘true’, CV_t_^2^ as measurement of heterogeneity of pulmonary perfusion. We hypothesized that statistical characteristics of multiple measurements provide the theoretical background to estimate *CV*_t_^2^. Additionally, we aimed at a validation of the method to estimate *CV*_t_^2^ using a phantom with a known structural heterogeneity, and to demonstrate advantages and repeatability of the estimation of *CV*_t_^2^ using our method.

## Methods

### Theoretical Basis

Measured values (*x*) including a measurement error can be described as the sum of the true value (*x*_τ_) and the error (*e*)

$$x=x_{\tau }+e$$ 1

For errors that are random realizations of a normal distribution (*N*) with an expectation value of zero and a known standard deviation (σ), the measured value can be described as

$$x=x_{\tau }+N(0,\sigma )$$ 2

Multiple measurements of *x* while *x*_τ_ is constant, e.g. at a steady state, allow the calculation of a mean value ($\bar{x}$) with a statistical variability known as the standard error of the mean, and defined as the standard deviation of the multiple measurements divided by the square root of the number of measurements (*n*):

$$\bar{x}=x_{\tau }+N(0,\sigma )/\sqrt{n}$$ 3

Note that the actual error of the mean value remains random depending on the random errors of the individual measurements.

In PET imaging, the basic characterization of a measured value with an error (Eq 1) applies to the activity sampled in individual voxels, forming a three-dimensional data matrix. The measured activity *A*(*j*) of voxel *j* is proportional to the cumulative count of registered decay events over the time of acquisition (*T*) of the PET scan. The measurement error of that mean activity can be described as the standard error of a mean value. For a PET scanner’s low range of activity having fully recoverable dead-time correction, it has been shown that the *n* affecting the noise level is proportional to the activity *A*_T_ in the field of view of the PET scanner [26]. A generalization of that effect for conditions with differences in acquisition time *T* yields that *n* is proportional to the product of activity and time:

$$A(j)=A_{t}(j)+N(0,\sigma )/\sqrt{n}$$ 4

with *n* ∞ *A*_T_ * *T*. Note that *n* is different from the registered number of decay events since PET requires detection of two photons per decay event, corrections for attenuation by the tissue of the body and for other factors. Also, PET scans show a radial dependence in the spatial distribution of the error’s magnitude with larger errors at the center [26]. However, this aspect is not critical for the final result as long as the errors of the individual voxels are random numbers from a distribution with an expectation value of zero (*N*(0,.)). That condition means that for *n* approaching infinity, e.g. very long acquisition times, *A*(*j*) approaches *A*_t_(*j*) even if the magnitude of noise is not homogeneous among voxels.

The variance of the voxel activities *A*(*j*) among all voxels of a region of interest (*j* ∈ ROI) of a PET scan is

$$\text{var}(A(j))=\text{var}(\;A_{t}(j)+e(j)/\;\sqrt{n})$$ 5

with *e*(*j*) = *N*(0,σ). Note that throughout the paper the variance is always calculated for *j* ∈ ROI, but we omitted it to simplify the notation. The *CV* ^2^ among voxels of an ROI of a PET scan (*CV*_s_^2^) is the variance among *A*(*j*) over the square of the mean of *A*(*j*) (${\bar{A}}^{2}$)

$$CV_{s}{}^{2}=\text{var}(\;A(j))/{\bar{A}}^{2}$$ 6

Since the realizations of the random errors *e*(*j*) are from a normal distribution with a mean of zero *N*(0,.), the terms *A*_t_(*j*) and *e*(*j*) are uncorrelated, and the overall variance of the PET scan can be described as the sum of the variances of *A*_t_(*j*) and *e*(*j*). Note that the validity of the lack of correlation between signal and noise is essential for the application of the method in experimental data, and a common requirement of parameter estimation methods. The separation of the two terms yields the heterogeneity of the noise-free ‘true’ activity ($CV_{t}{}^{2}=\text{var}(\;A_{t}(j))/{\bar{A}}^{2}$) and the noise of the PET scan

$$CV_{s}{}^{2}=CV_{t}^{2}+1/n\cdot \text{var}(\;e(j))/{\bar{A}}^{2}$$ 7

with *e*(*j*) = *N*(0,σ). Also, $1/\sqrt{n}$ is a constant among the random errors so that it can be moved outside of the variance and becomes 1/*n*. Furthermore, Eq (7) shows that *CV*_s_^2^, derived from the activity values of a single frame, are overestimated due to imaging noise unless the number of counts is infinite.

Dynamic PET scans are sequences of time frames to assess the kinetics of tracer distributions. For example, we analyzed for this study a selection of the first 10 frames of dynamic PET scans after ^13^NN bolus injection, as described below, with frame durations of 8 x 2.5 s, and 2 x 5 s. Under steady state conditions of the spatial tracer distribution, the concentration in each voxel is theoretically constant over time. Frames with a longer duration have a larger *n*, and lower noise and *CV*_s_^2^. Multiple frames indexed *k* that may vary in 1/n and *CV*_s_^2^ can be used to estimate a linear regression:

$$CV_{s}{}^{2}(k)=CV_{t}^{2}+1/n(k)\cdot b+e_{r}$$ 8

where *e*_r_ is the residual error of the regression. The linear regression of *CV*_s_^2^(*k*) over 1/*n*(*k*) yields estimates for a y-intercept *CV*_t_^2^, and a slope *b*. The slope replaces the term of the random errors so that details of the random errors are not relevant as long as their characteristics are constant during the time period of the selected frames of a dynamic PET scan. To increase the range of 1/n and *CV*_s_^2^ values for the linear regression, we merged combinations of the selected frames. For *M* frames selected from the PET scan, a list *l*(*k*) with all possible combinations has the length *k*_max_ = 2^*M*^ − 1. This list includes for the element *k*_max_ all frames, e.g. *M* = 3 results in the list *l* = {1; 2; 3; 1 2; 1 3; 2 3; 1 2 3} with *l*(*k*_max_) = {1 2 3}. For each item of *l*(*k*), which is a combination of frames, the merged time *T*(*k*) of the combination was calculated as sum of the original frames’ time *T*_o_(*i*)

$$T(k)=\sum_{i\in l(k)}T_{0}(i)$$ 9

and the merged activity *A*(*j*,*k*) of each voxel *j* was derived from the original frames *A*_o_(*i*,*j*) as time-weighted mean activity

$$A(j,k)=\sum_{i\in l(k)}\left(A_{0}(i,j)\cdot T_{0}(i))/T(k\right)$$ 10

with *j* ∈ ROI, and *k* = 1. *k*_max_. The two sets of parameters for the linear regression were calculated with

$$n(k)=T(k)\cdot {\bar{A}}_{ROI}(k_{\text{max}})\cdot {\bar{A}}_{t}(k)/{\bar{A}}_{t}(k_{\text{max}})$$ 11

$$CV_{s}^{2}(k)=\text{var}(A(j,k))/{\bar{A}}_{ROI}{}^{2}(k_{\text{max}})\cdot {\bar{A}}_{t}{}^{2}(k_{\text{max}})/{\bar{A}}_{t}{}^{2}(k)$$ 12

where var(*A*(*j*,*k*)) is the variance of activity within the ROI (*j* ∈ ROI) for each frame *k*, $\bar{A}$_t_(*k*) is the total mean activity of the frame, and $\bar{A}$_ROI_(*k*_max_) is the mean activity of the ROI using all selected frames so that the most reliable statistical estimate of the mean activity is obtained. The term $\bar{A}$_t_(*k*_max_) / $\bar{A}$_t_(*k*) corrects for fluctuations during steady state conditions, and increases the robustness of the estimation.

The contribution of noise to *CV*_s_^2^(*k*) can be derived from (6) as

$$CV_{n}^{2}(k)=CV_{s}^{2}(k)-CV_{t}^{2}(k)$$ 13

### Phantom PET Scan and Analysis

**Fig 1:**
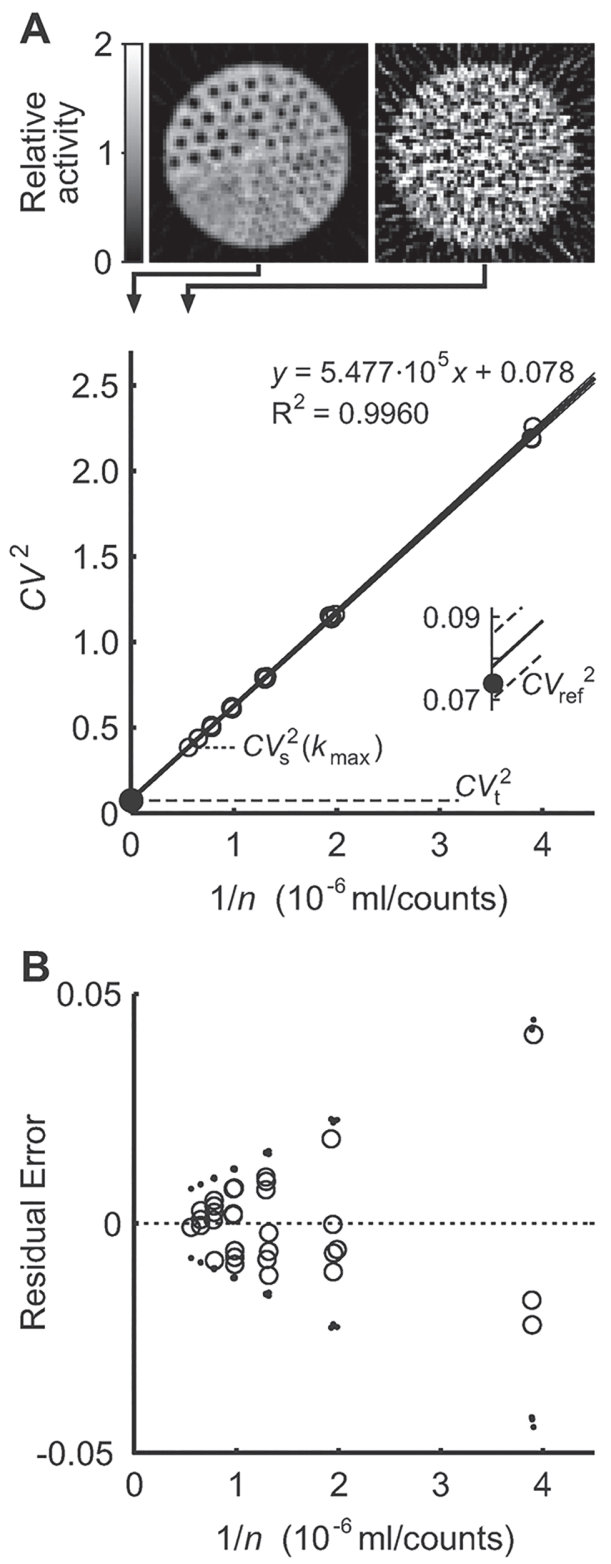
Estimation of noise-free heterogeneity and evaluation using a PET emission scan of a Jaszczak phantom filled with a solution of ^18^F-FDG and water. **A**: The *CV*_s_^2^(*k*) data (○) for the linear regression were derived from five frames (3 x 5 s, 2 x 10 s). *CV*_s_^2^(*k*_max_) showed the lowest heterogeneity for the merged frame with total acquisition time of 35 s. The reference heterogeneity (•) of a 573 min PET scan to minimize noise lay within the narrow 95% confidence interval of the estimated noise-free heterogeneity (see inset). Representative slices of the reference scan (top left) and at *k*_max_ (top right) show the difference in noise in the PET images. Note that the confidence interval is included but so close to the regression line that it is very difficult to distinguish and required the inset for details. **B**: Residual errors of the linear regression (○) were unbiased relative to the prediction of the linear regression, and symmetrically distributed within the standard error (95% confidence interval) of the variance estimates (▪). Note that the estimated standard errors of each *CV*_s_^2^(*k*) are plotted relative to the values predicted by the linear regression so that they show the confidence interval around the predicted variance. The cone shapes of both *e*_r_ and the standard errors of variances pointing to zero residual errors for an infinite number of counts illustrate the theoretical concept of the estimation method that the standard error of the variance estimates decreases with an increase in counts.

For experimental evaluation of the estimation method, a Jaszczak phantom (Fig 1A) was filled with a solution of ^18^F-FDG (total activity 189 MBq) and was placed in the multiring whole-body PET scanner (Scanditronix PC4096, General Electric Medical Systems) with its cold rod inserts in the center of the field of view. The PET scanner acquired (in stationary mode) 15 transverse cross-sectional slices of 6.5-mm thickness providing 3-dimensional information over a 9.7-cm-long cylinder. We acquired a dynamic PET scan including 53 frames with a total acquisition time of 573.3 min: 4 x 2.5 s, 4 x 5 s, 5 x 10 s, 2 x 60 s, and 38 x 900 s. The timing of the early frames is equal to the frame durations of the pulmonary ^13^NN PET scans described below so that conditions are comparable. A transmission scan was performed using 10 min acquisition with a rod source of ^68^Ge rotating around the phantom. The transmission scan was used to correct for photon attenuation by the phantom. The ROI for the phantom’s chamber was defined by applying a threshold at 50% peak activity to the ^18^F-FDG emission scan and including all cold rod inserts.

### Pulmonary Perfusion Imaging and Analysis

To assess differences between *CV*_t_^2^ and *CV*_s_^2^ in PET scans of pulmonary blood flow, we analyzed in total 38 dynamic PET scans. These scans were previously acquired from 20 healthy subjects and 1 COPD subject as part of physiological studies approved by the Human Research Committee of the Massachusetts General Hospital [4,5,27,28].

To evaluate the reproducibility of the perfusion *CV*_t_^2^ estimates, we compared multiple PET scans of the same subject. These were available for 4 control subjects and the COPD patient (3 consecutive scans were acquired at approximately 30 minute-intervals). They had been part of a protocol in which isocapneic hyperpnea was induced shortly after the second scan, but the ventilatory pattern had returned to baseline before the third PET scan. One PET scan from this group could not be used because the duration of steady state plateau in ^13^NN kinetics was too short.

In these studies, pulmonary blood flow was measured by a single bolus injection of ^13^NN [29–31]. Briefly, the single bolus method consists on injecting a bolus of saline with dissolved ^13^NN (average activity = 750 MBq) into a peripheral venous catheter at the beginning of an apnea period. When the ^13^NN tracer reaches the lungs it rapidly diffuses into the alveolar gas phase, where it remains during the apnea period without significant change in concentration. During the last frames of the apnea period, when the tracer activity shows a steady state, the spatial distribution of tracer activity is proportional to the regional blood flow and allows the measurement of the *CV*_s_^2^ of pulmonary blood flow. The imaging sequence during the apnea period consisted of 10 frames with a total time of 30 s (8 x 2.5 s, 2 x 5 s). Other frames following the apnea period in the original PET scans are not relevant for this analysis.

Transmission scans were obtained before radioactivity administration to correct for photon attenuation caused by body tissues, and to delimit ROIs for the lungs, using a threshold at 50% density of water. The acquisition consisted of a 10 min scan with a rod source of ^68^Ge rotating around the body. After correction of detector sensitivity, scattering, and attenuation, the PET emission scans were reconstructed using a filtered backprojection with a Hanning filter of 6.4 mm width. The resulting PET scans consisted of an interpolated matrix of 128 x 128 x 15 voxels of 4 x 4 x 6.5 mm size each. The transmission scans were reconstructed with a filter width of 11.5 mm.

Specific frames of the PET scans were selected to estimate the noise-free heterogeneity. For the experimental evaluation, the frames 6 to 10 of the phantom PET scan were used to match the conditions of human studies for number of frames, acquisition time, and activity. For the human studies, the frames were selected manually using the time activity curve of the whole field PET scan to identify the period with constant activity during apnea. Each study needed to have at least two, but ideally three or more, frames with approximately equal activity. For both the phantom and the human studies, the decomposition of variances was performed to estimate noise-free heterogeneity using the proposed method implemented in MATLAB (MathWorks, Natick, Massachusetts). The residual error *e*_r_^2^ between measured *CV*_s_^2^(*k*) and the value predicted by the linear regression was calculated for each frame *k* as *e*_r_(*k*)^2^ = *CV*_s_^2^(*k*) − *CV*_t_^2^ − 1/*n*(*k*) *b*. In order to evaluate the characteristics of *e*_r_(*k*)^2^, standard errors of the variance estimates (95% confidence interval) [32] were calculated and plotted relative to the value predicted by the linear regression. It was expected that the majority of *e*_r_ would be within the range the standard error of the variance estimates relative to the predicted value.

## Results

### Phantom PET Scan

Linear regression (Eq 8) yielded an estimated noise-free heterogeneity of *CV*_t_^2^ = 0.078 with R^2^ = 0.996 (Fig 1A). Compared to the reference heterogeneity *CV*_ref_^2^ = 0.074 of the cumulative PET scan with a total acquisition time of 573.3 min, the *CV*_t_^2^ estimate was 5.4% higher than *CV*_ref_^2^. The 95% confidence interval of *CV*_t_^2^ was ±10% and very narrow relative to the range of the linear regression (Fig 1A). The accuracy of that estimate is remarkable given the large contribution of noise to *CV*_s_^2^(*k*_max_). Also, *CV*_ref_^2^ lay within the confidence interval of the estimated *CV*_t_^2^ (Fig 1A, inset). The residual errors of the linear regression showed an unbiased symmetric wedge-shaped distribution and all errors lay within the 95% confidence intervals of the estimated variances relative to the regression line (Fig 1B).

To evaluate the *CV*_t_^2^ of ROIs relative to other ROIs and to test for a bias, the circular ROI of the phantom chamber was divided in two sub-ROIs: the central circle and the surrounding ring each occupying 50% of the total ROI. The heterogeneities and the mean values of the two sub-ROIs were used to predict the theoretical *CV*_t_^2^ of the unified ROI. The error between predicted and measured value was *ΔCV* ^2^ < 1.5% demonstrating the consistency of the estimated heterogeneity.

### Pulmonary Perfusion Imaging

**Fig 2:**
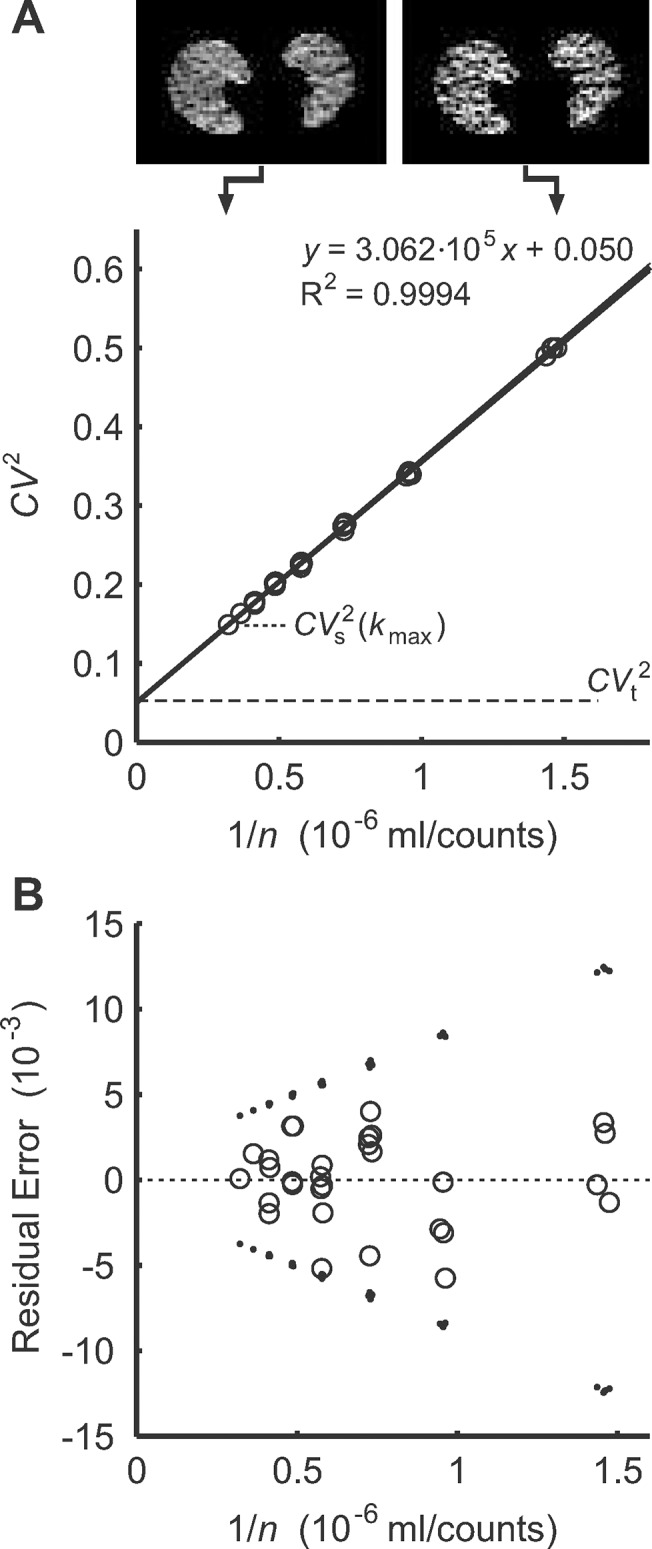
Example of an estimation of noise-free heterogeneity *CV*_t_^2^ of pulmonary blood flow in a subject. **A**: Linear regression to estimate *CV*_t_^2^ and examples of two selected slices illustrating the difference in the noise levels between a single frame (right) and the merged frame *k*_max_ with the longest acquisition time (left). Note that the 95% confidence interval is included but visually difficult to distinguish from the regression line. **B**: Residual errors of the linear regression (○) were unbiased and symmetrically distributed within the standard error (95% confidence interval) of the variance estimates (▪). This behavior was very similar to the characteristics of the variance in the phantom image (Fig 1).

For dynamic PET scans of pulmonary perfusion, *CV*_t_^2^ was estimated from a minimum of two and a maximum of seven frames of the PET scans comprising in total an acquisition time between 7.5 and 30 s with a constant tracer distribution during the apnea period. The linear regression to estimate the noise characteristic and *CV*_t_^2^ yielded excellent correlation coefficients similar to that in the PET phantom study (Fig 2A). The residual errors of the linear regression had in all PET scans a wedge-shaped distribution and the majority of errors lay within the 95% confidence intervals of the variance estimates relative to the regression line (Fig 2B).

**Fig 3:**
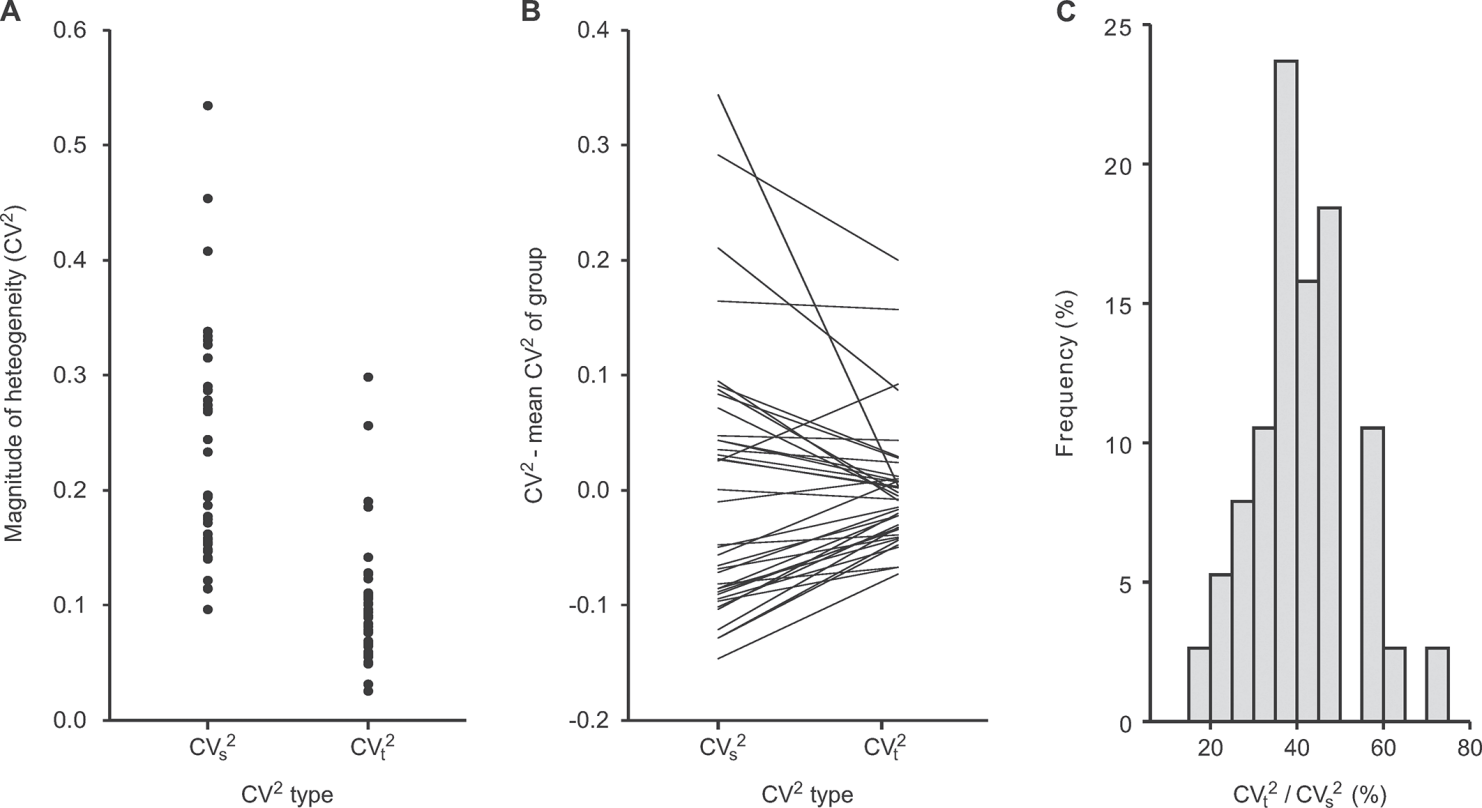
Comparison between estimates of noise-free heterogeneity (*CV*_t_^2^) in pulmonary perfusion and the corresponding overall heterogeneity of PET scans including noise (*CV*_s_^2^). **A:** Paired magnitudes of *CV*_s_^2^ and *CV*_t_^2^ of perfusion. **B:** Comparison of *CV*_s_^2^ and *CV*_t_^2^ relative to each parameter’s mean showing that the noise included in *CV*_s_^2^ affected the order of values compared to *CV*_t_^2^. That difference in the order of values would for example affect rank sum tests and result in a higher statistical power for tests performed with *CV*_t_^2^ compared to *CV*_s_^2^. In general, noise affecting a parameter lowers its accuracy for comparisons. **C:** *CV*_t_^2^ over *CV*_s_^2^ as relative fraction of the noise-free heterogeneity compared to the overall heterogeneity of PET scans.

When analyzing the results from all pulmonary perfusion scans (n = 38), the average noise-free heterogeneity was *CV*_t_^2^ = 0.10 with a range from 0.03 to 0.30, while the average overall heterogeneity of the PET scans was *CV*_s_^2^(*k*_max_) = 0.24 with a range from 0.10 to 0.59 (Fig 3A). *CV*_t_^2^ normalized by *CV*_s_^2^(*k*_max_) as relative fraction of the noise-free heterogeneity compared to the heterogeneity of the PET scan was on average 41.5% with a range from 17.8% to 71.2% (Fig 3C). The estimated *CV*_t_^2^ were correlated with their respective *CV*_s_^2^ (r = 0.75, p<0.001) indicating that differences in the heterogeneity of PET scans including noise were representative of differences in the noise-free heterogeneity. However, for statistical comparisons between different conditions or groups it is important to know if *CV*_s_^2^ may be sufficient to detect differences in comparison to tests performed with *CV*_t_^2^. A pair-wise visualization of *CV*_s_^2^ and the noise-free *CV*_t_^2^ of each pulmonary perfusion scan relative to each parameters mean value shows that the noise component was variable among the PET scans and caused substantial scattering of *CV*_s_^2^ values in comparison to the order of the noise-free *CV*_t_^2^ values, suggesting that *CV*_t_^2^ is more reliable for comparisons and statistical tests than *CV*_s_^2^ (Fig 3B).

**Fig 4:**
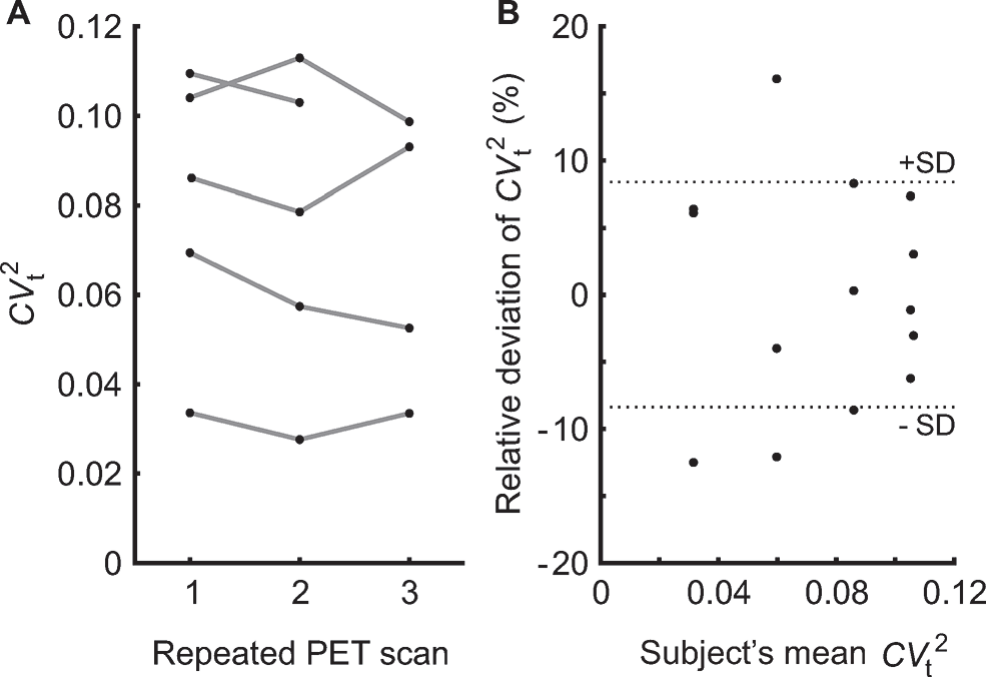
Evaluation of the reproducibility of the estimation method using multiple PET scans of pulmonary perfusion in five subjects to estimate noise-free perfusion heterogeneity (*CV*_t_^2^). **A:** Estimated *CV*_t_^2^ showing the intra-subject variability (connected data points) and the inter-subject differences. **B:** Relative deviation of *CV*_t_^2^ from each subject’s mean vs. the subject’s mean *CV*_t_^2^ of repeated PET scans shows the normalized intra-subject variation among repeated scans that was used to characterize reproducibility. The standard deviation (SD) was 8.4%.

**Fig 5:**
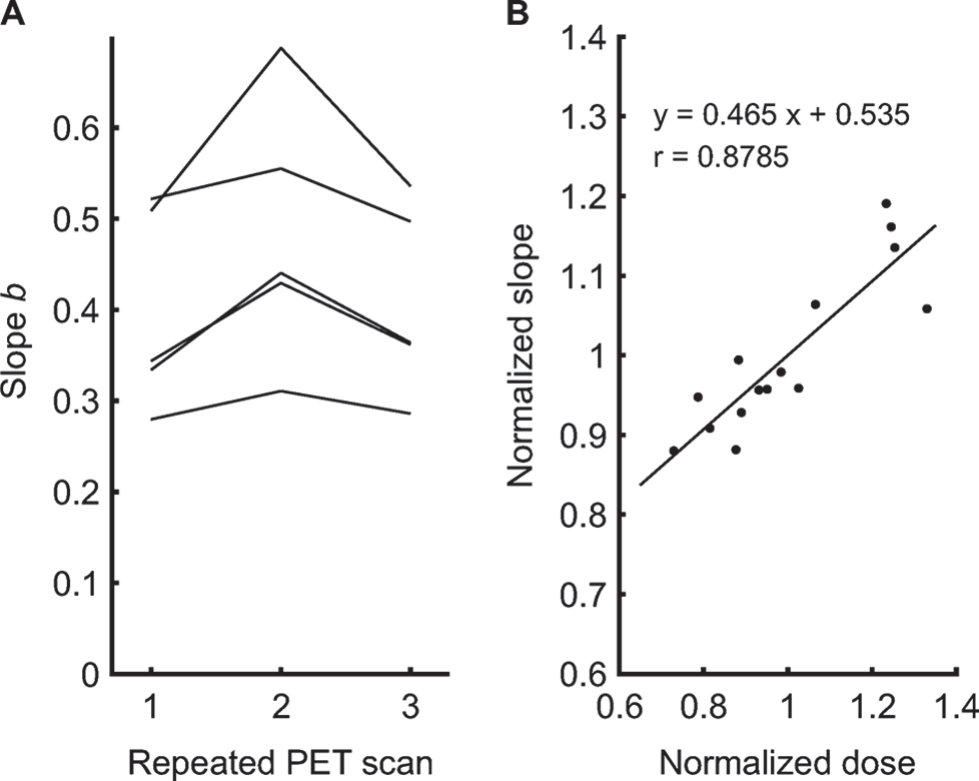
Effects on the noise characteristic of PET scans. **A:** Estimated slopes *b* of the linear regression between *CV*_s_^2^ and 1/n in five subjects submitted to multiple PET scans. The substantial inter-subject variability compared to the intra-subject variability was consistent with the expectation that differences in the amount of tissue surrounding the lungs cause differences in attenuation and, thus, in the actual number of detected decay events. **B:** Linear regression between the estimated slopes *b* normalized by each subject’s mean slope and the injected ^13^NN doses normalized by each subject’s mean dose.

A separate analysis was performed in the subgroup submitted to multiple PET scans. The evaluation of the reproducibility of *CV*_t_^2^ estimates showed a standard deviation of 8.4% for the *CV*_t_^2^ estimates of repeated PET scans relative to the subject’s mean. 50% of the *CV*_t_^2^ had a relative deviation from the subject’s mean of less than 6.3%, and the maximum deviation was 16.1% (Fig 4). Also, there was no difference among the *CV*_t_^2^ obtained from the three PET scans when the Paired Samples t-test was performed. We found also that inter-subject differences had a larger effect on the slope *b* of the linear regression between *CV*_s_^2^ and 1/n than the intra-subject variations (Fig 5A), and that relative differences in *b* were correlated with relative differences in the injected dose (Fig 5B).

## Discussion

In this study we demonstrated by theoretical analysis, a PET scan of a phantom, and the evaluation of pulmonary perfusion scans in human subjects that noise-free spatial heterogeneity can be estimated based on a relationship between the heterogeneity of a measured data set (*CV*_s_^2^) and the conditions of measurement (1/n) applied to dynamic PET scans. Additionally, we found that the noise-free heterogeneity *CV*_t_^2^ was substantially lower than the corresponding heterogeneity of the PET scan including noise, that the noise-free *CV*_t_^2^ estimates have a very good reliability, and that there were subject and dose related effects on the slope of the regression between *CV*_s_^2^ and 1/n.

The theoretical basis of the described method to estimate noise-free *CV* ^2^ shows that applications of the estimation method are in principle not limited to PET imaging. The method may be used for example to estimate the noise-free variance or *CV* ^2^ of a parameter in a group of subjects, to compare the variance of two groups, or to characterize the spatial or temporal heterogeneity among points of measurement. The described estimation of the noise-free variance depends on the following assumptions: 1) for each data point, multiple measurements are acquired while the observed variable *x*_t_ is at a steady state, and 2) each individual measurement has a random error from a distribution with an expectation value of zero added to *x*_t_. The second requirement that errors are random and have an expectation value of zero is relatively universal for parameter estimation. It describes the problem that any expectation value different from zero results in a correlation of the error with the signal, and causes a bias of parameter estimates. For example, Eq 2 discriminates between a steady state parameter and a random deviation with an expectation value of zero. Any constant offset originating from the measurement error would result in a bias of the steady state parameter. Also, note that the assumption of an expectation value of zero should be true for the time frame of the data acquisition in case there are fluctuations over time. Errors including very low frequencies or drift may require additional data processing to avoid expectation values different from zero. For dynamic PET imaging, the primary source of errors is the randomness of the radioactive decay process. Other sources include random coincidences, scattering, detector inhomogeneity, and attenuation correction. The last two have the potential to cause errors that are constant among the frames of a dynamic PET scan so that they could lead to error distributions with expectation values different from zero and, thus, biased estimates of *CV*_t_^2^. The magnitude of the random errors in the measured activity *A*(*j*) of voxel *j* was described by the constant σ (Eq 4) although there is a radial dependence in PET scans [26] and σ(*j*) could be used to characterize variations in the random distributions *N*(0,σ(*j*)) among voxels. For simplicity, we chose σ because we expected that estimates of *CV*_t_^2^ as the constant parameter of the linear regression are independent of variations in σ(*j*), and that the slope is affected by the average σ(*j*) within an ROI. Also, our results demonstrate that the estimated *CV*_t_^2^ of the phantom PET scan matched the reference value *CV*_ref_^2^ despite the variation of σ(*j*), and that the estimates of sub-ROIs were consistent with the global estimate of the phantom although the two sub-ROIs covered different radial distances from the center so that the radial dependence of the errors must have caused differences in average σ(*j*) between the two sub-ROIs.

With the given assumptions, our approach could also be used to estimate the noise-free variance of a parameter in a group of subjects if multiple measurements can be performed in each subject or point of measurement while the parameter is at a steady state. *CV*_t_^2^ of the parameter would be estimated using the sequential measurements similar to sequential frames in dynamic PET scans. However, if an application aims to determine heterogeneity in time and needs to eliminate random sampling errors related to spatial fluctuations of the parameter it may be necessary to perform multiple simultaneous measurements at different locations, e.g. using a network of sensors, rather than different time points.

The theoretical analysis has led to a mechanistic understanding of the statistical cause-effect relationship between the *CV*_s_^2^ and 1/*n*. This analysis was inspired by a previous study showing in PET scans of a phantom that the two parameters were correlated [25]. The linear regression to estimate noise-free heterogeneity was conducted for spatial tracer distributions that were temporarily in steady state, using multiple frames of a dynamic PET scan. The temporarily constant distribution of tracer activity eliminates the potential influence of non-linear relations between activity, registered counts, and variance [34], which could make the linearity between *CV* ^2^ and 1/*n* less valid. It is important to note that our use of the term noise-free is limited to random fluctuations over time. Systematic errors affecting the expectation values of voxels, such as those introduced by errors in the transmission scan, require different approaches than multiple measurements over time. Our analysis of the phantom PET scan allowed a comparison of *CV*_ref_^2^ with *CV*_s_^2^(*k*_max_), the best estimate of the PET scan for conditions comparable to our pulmonary perfusion scans (Fig 1A). The differences show that the contribution of systematic errors to the *CV*_t_^2^ must have been very small compared to the random fluctuations over time (Fig 1A).

The experimental validation using a phantom with a known structure provided two important results for the validation of *CV*_t_^2^. First, the evaluation showed that *CV*_ref_^2^ lay within the very narrow 95% confidence interval at the y-intercept of an excellent correlation (Fig 1A). Second, the residual errors of the linear regression were not only symmetric but also centered around zero and within the 95% confidence intervals of *CV* ^2^ estimates (Fig 1B). Note that the confidence interval shows the uncertainty of the estimate similar to the well-known concept of the standard error of an estimated mean value. The estimate is expected to be with a certain probability within the confidence interval. These characteristics of the residuals indicate that the described theoretical basis applies to PET imaging, that the estimates of *CV*_s_^2^ from PET scans vary around the true value similar to mean values of a random sample, and that this variation decreases with larger *n* similar to the standard error of the estimates of the mean. The cone shape of the 95% confidence interval of the *CV*_s_^2^ estimates in regard to 1/n is related to the linear relationship the standard error of mean estimates describes between the variance of mean estimates and 1/n.

Validation of heterogeneity measurements can be a major challenge because the resolution of an image, or a measurement in general, typically, has an effect on the estimated variance [17]. In fact, the relationship between resolution and *CV* ^2^ is frequently used in fractal analysis. A dependence on resolution means that a comparison of *CV* ^2^ values between studies requires at least comparable imaging techniques and resolutions for the measurements, which was the case for our validation using the Jaszczak phantom and the same settings for PET image reconstruction as for the human studies. A validation study of the PET scanner’s resolution showed for distances of 0−20 cm from the center a full width at half maximum (FWHM) of 6−7 mm [33]. It is possible that systematic spatially inhomogeneous errors of the imaging process caused an increase in *CV*_ref_^2^ compared to an ideal PET scan of the phantom but there are no signs of substantial artifacts in the reference PET scan (Fig 1A).

The results of pulmonary perfusion imaging showed that the estimated *CV*_t_^2^ were reliable even when only two frames with stable measured activity were used for the estimation. Moreover, the fact that *CV*_t_^2^ and *CV*_s_^2^ were highly correlated but the corresponding value pairs had significantly different positions in the ranking of *CV*_t_^2^ and *CV*_t_^2^ highlights the distortions caused by errors of measurement in *CV*_s_^2^ as well as the importance of using techniques to estimate *CV*_t_^2^ when studying heterogeneity of physiological processes. Finally, when it comes to the reproducibility of our method, it is notable that individual *CV*_t_^2^ were within 16% of the mean value for each subject, with 50% of the sample being within 7% of the mean value. Importantly, there was no difference among the *CV*_t_^2^ obtained from the 3 tests when the Paired Samples t- test was performed. The slope of the linear regression between *CV*_s_^2^ and 1/*n* should be constant if the origin of the noise is not variable and *n* is the number of measurements included in the averages of the individual points of measurement. In pulmonary PET imaging, however, tissue surrounding the lungs scatters some of the photons decreasing the number of events the PET scanner counts. The activity inside the lung is estimated by correcting for tissue attenuation. That means that differences in the attenuation among subjects were expected to affect the relationship between *CV*_s_^2^ and 1/*n*. In fact, our findings of differences among the slopes *b* confirm an effect of the subject on the relationship between *CV*_s_^2^ and 1/*n*, and the largest differences in slope could be attributed to an effect of the subject on the slope (Fig 5A). Surprisingly, relative changes in the injected dose were correlated with relative changes in slope (Fig 5B). Both parameters were normalized for each individual by the subject’s mean of the parameter. The correlation may be caused by residual activity in a device near the PET scanner that was used for processing and injection of ^13^NN, but the effect is not relevant for the estimation of the noise-free *CV* ^2^ since the slope is in our method estimated for each PET scan.

Measuring the heterogeneity of physiological processes can be crucial for the understanding of both normal function and pathological changes [4,5,7,35,36], and can be a biomarker for disease [6]. For pulmonary perfusion, we demonstrated the lower estimates of noise-free heterogeneity compared to the overall heterogeneity in PET scans. Also, the noise affected differences in *CV*_s_^2^ values compared to *CV*_t_^2^ altering the order of values (Fig 3). Rank sum tests, for example, would be directly affected by that difference so that *CV*_t_^2^ provides a higher statistical power than *CV*_s_^2^. In general, the noise included in *CV*_s_^2^ decreases the accuracy of differences among groups compared to *CV*_t_^2^ and the statistical power to discriminate for example healthy subjects from patients with a disease. More reliable estimates of noise-free *CV*_t_^2^ compared to *CV*_s_^2^ may also be relevant for the heterogeneity of other physiological parameters including for example metabolic activity in organs or related to inflammatory processes, myocardial metabolism or perfusion, or for tumors.

Noise can be a major limitation in nuclear imaging especially for quantitative measurements and statistical tests when imaging noise increases the variability of the data. Our method to estimate noise-free *CV* ^2^ using multiple frames of a dynamic PET scan has the advantage that it does not require access to the sinogram, which is used for other methods. Furthermore, the contribution of noise to the overall variance of an ROI is, in contrast to static structural heterogeneity, a relevant statistical measure of uncertainty.

The method to estimate noise-free variances was developed and tested using a PET scanner in 2D acquisition mode, and filtered backprojection to reconstruct the images. In 3D mode, the method may be affected by the lower axial resolution of the 3D mode [26,37] or by changes of local variance in axial direction [26,38]. But both effects are unlikely to affect the linear relationship between *CV*_s_^2^ and 1/*n* so that the estimation method should work for 3D acquisition mode. Note that differences in resolution may affect the *CV*_t_^2^ as discussed above. Iterative reconstruction methods may also affect the relationship between *CV*_s_^2^ and 1/*n*, and it is unclear if the relationship is linear for all reconstruction methods. Dahlbom has shown very similar noise characteristics for filtered backprojection and ordered subset expectation maximization (OSEM) in phantoms with homogeneous tracer distribution [14], but it is unclear if the noise characteristics would be different for inhomogeneous tracer distributions.

Despite having succeeded in developing a method that is easily implemented in the process of assessing heterogeneity of different biological events, our study has several limitations. First and most importantly, the method relies on the assumption of stable activity throughout a certain period of time (breath hold) in order to correctly estimate *CV*_t_^2^. However, many subjects on the multiple measurements analysis were not able to hold their breath long enough to obtain a discernible plateau on the kinetics of ^13^NN, so that only two frames could be used to calculate the perfusion heterogeneity in most cases. Independent of that, the method proved to be both reliable and reproducible. Second, the protocols from which the data was acquired had not been designed for the reproducibility of *CV*_t_^2^ so that they could have inadvertently affected our results. For instance, the subjects in the multiple-measurement group had a period of hyperpnea induced with carbon dioxide rebreathing shortly after each PET scan. Nevertheless, we believe that this intervention was unlikely to have had any long-lasting effects on pulmonary perfusion since ventilation had returned to baseline in all subjects by the time the next PET scan was performed.

For future applications in dynamic PET or SPECT scans without a period of unchanging tracer distribution, it should be possible to simulate parallel acquisition of multiple frames by randomly or systematically decreasing the counts in list mode data. The duration of such virtual frames would be identical but with different numbers of counts *n* and, thus, different *CV*_s_^2^ so that they can be used to estimate the noise-free *CV*_t_^2^.

In summary, noise-free variances or coefficients of variance as a measure of spatial heterogeneity can be estimated using multiple measurements in dynamic PET scans. Experimental data from a phantom and human studies showed the validity of the method and that the estimated noise-free heterogeneity may be substantially lower than the overall heterogeneity in PET scans. Also, the theoretical analysis revealed the principle of the method, which has potential for applications in other fields. Our method has proven its efficiency in imaging analysis and may help to improve quantitative studies of structural and functional heterogeneity or conditions affecting that heterogeneity.
